# Anesthetic management for mastectomy and total hysterectomy in a 49-year-old woman with type 1 sialidosis: a case report

**DOI:** 10.1186/s40981-021-00425-z

**Published:** 2021-03-04

**Authors:** Tomonori Furuya, Masumi Itagaki, Nami Sugaya, Ryoji Iida, Takeshi Maeda, Takahiro Suzuki

**Affiliations:** 1 Department of Anesthesiology, Nihon University Hospital, 1-6 Kanda, Surugadai, Chiyoda-ku, Tokyo, 101-8309 Japan; 2 Department of Anesthesiology, Nihon University School of Medicine, 30-1 Oyaguchi, Kamimachi, Itabashi-ku, Tokyo, 173-8610 Japan

**Keywords:** Sialidosis, Lysosomal storage disease, Total intravenous anesthesia, Epidural anesthesia

## Abstract

**Background:**

Sialidosis is an autosomal recessive glycoprotein storage disorder, caused by neuraminidase deficiency which leads to abnormal intracellular accumulation and urinary excretion of sialylated oligosaccharides, resulting in various morphological and functional disorders. Only a few reports have described the anesthetic managements of patients with sialidosis.

**Case presentation:**

A 49-year-old woman with type 1 sialidosis suffered from all limb contractures, an ocular cherry-red spot, and myoclonic seizures of the limbs. She had been cognitively normal. She was separately scheduled for mastectomy under total intravenous anesthesia and total hysterectomy under combined general and epidural anesthesia uneventfully.

**Conclusions:**

Our patient with type 1 sialidosis received both general and epidural anesthesia uneventfully. Anesthesiologists should carefully assess patients with sialidosis and give careful consideration to individually tailored anesthetic managements.

## Background

Sialidosis is an autosomal recessive glycoprotein storage disorder, caused by neuraminidase deficiency which leads to material rich in sialic acid to accumulate in various tissues and organs, leading to morphological and functional disorders.

Type 1 sialidosis is characterized by the development of ocular cherry-red spots, decreased visual acuity, impaired color vision, debilitating generalized myoclonic seizures, and gait abnormalities [1]. For a very rare disease, only two reports have described anesthetic management of patients with sialidosis [2, 3]. Here, we present anesthetic managements for a 49-year-old woman with type 1 sialidosis, who was separately scheduled for mastectomy and total hysterectomy.

## Case presentation

A 49-year-old woman with type 1 sialidosis (weight 49.2 kg, height 158.0 cm) had developed gait disorders at the age of 11 years and was diagnosed with type 1 sialidosis by genetic testing. The muscle weakness progressed to a manual muscle testing of 2/2. Therefore, her all limbs were contracted close to functional position and limitation of joint range of motion was developed. Therefore, she required complete assistance in her daily life activities from the age of 18 years. She had been wheelchair-bound when going out and bedridden in her home. She was able to swallow minced meal with full assistance and used to receive powdered medication through a gastrostomy.

She had been cognitively normal and could adequately understand our explanation about the proposed anesthetic managements. However, articulation disorders precluded smooth communication. Additionally, an ocular cherry-red spot was confirmed, and she suffered from progressive visual impairment. She was receiving levetiracetam and sodium valproate as prophylactics against myoclonic seizures of the limbs, and she had not experienced any seizures for the last few years.

She presented neither facial dysmorphism nor scoliosis. Magnetic resonance imaging did not reveal any intraspinal malformation. Orofacial examination revealed neither deformations nor growths such as occult tracheal granulomas in the oral cavity. She had neither neck extension restriction nor temporomandibular joint stiffness. Oral evaluation also revealed a Mallampati class 1 airway. She had no history suggestive of either obstructive sleep apnea or recurrent pneumonia. Her blood analysis, electrocardiogram, echocardiography, and spirometry did not reveal any abnormality.

### Total mastectomy

At the age of 49 years, she was diagnosed with left breast cancer and was scheduled to undergo left total mastectomy. Anesthesia was planned as total intravenous anesthesia (TIVA) using propofol and remifentanil. She positioned supine on the operating table with all assistance. The gap between contracted limbs and the table was filled with some towels. Electrocardiogram, noninvasive blood pressure, percutaneous oxygen saturation, and bispectral index (BIS) were monitored during anesthesia.

With no premedication, rapid sequence induction was performed with fentanyl 50 μg and a targeted controlled infusion (TCI) of propofol. The initial effect-site propofol concentration was set at 2.0 μg/ml. After confirming loss of consciousness at an effect-site concentration of 0.8 μg/ml, rocuronium bromide 50 mg was administered before tracheal intubation. The endotracheal tube (inner diameter 7.0) was easily inserted using a McGrath^Ⓡ^ videolaryngoscope.

Anesthesia was maintained with a propofol infusion at the rate of 1.5–2.0 μg/ml and remifentanil infusion of 0.1–0.25 μg/kg/min to maintain BIS value between 40 and 60. Intravenous acetaminophen 750 mg was administered and local anesthetics (10 ml of 0.25% levobupivacaine) were injected at the surgical incision site. Intraoperatively, her blood pressure and heart rate stayed within 20% of preoperative values without special hemodynamic support. The surgery was uneventfully completed in 33 min and the blood loss was 1 ml.

Ninety seconds after administration of 100 mg of sugammadex, her train-of-four (TOF) ratio at the ulnar nerve recovered to 100%. She responds to her name being called with handshake, and spontaneous tidal volume was approximately 400 ml per inspiration at an effect-site propofol concentration of 0.5 μg/ml. Subsequently, her trachea was extubated 20 min after discontinuation of anesthetics. In the ward, acetaminophen was administered for analgesia as required. There were neither respiratory problems nor new neurological deficits during the postoperative course. She was discharged 7 days after surgery.

### Total hysterectomy

Three months after mastectomy, she presented with lower abdominal pain that was diagnosed with uterine fibroids and was scheduled to undergo open total hysterectomy. Anesthesia was planned as a combination of general and epidural anesthesia. Premedication was not prescribed. Preoperatively, an epidural catheter was uneventfully inserted via the T11-T12 intervertebral space. Next, the rapid sequence induction was performed with fentanyl 50 μg, a TCI of propofol and rocuronium bromide 50 mg. After smooth insertion of the endotracheal tube, anesthesia was maintained with a propofol infusion of 1.0–1.5 μg/ml and remifentanil 0.05–0.1 μg/kg/min. Patient-controlled epidural analgesia (PCEA) was initiated with 4 ml/h of 0.125% levobupivacaine, with a 3-ml bolus dose and 30-min lockout time, following administration of 3 ml 0.25% levobupivacaine via the epidural catheter.

The surgery was uneventfully completed in 3 h 7 min and blood loss was 212 ml. Hemodynamic stability was maintained without the need for cardiovascular support. After administration of 100 mg of sugammadex, the TOF ratio recovered to 100%. She emerged from anesthesia and her trachea was extubated 12 mins after discontinuation of the anesthetics. In the ward, PCEA alleviated her pain, with rescue acetaminophen given as needed. She was discharged on the 10th postoperative day after an uneventful postoperative course.

## Discussion

Sialidosis is an autosomal recessive glycoprotein storage disorder caused by neuraminidase deficiency resulting from a mutation in the neuraminidase gene, located on the 6p21.3 imprinted region [4]. These mutations express molecular heterogeneity, with a mixed variety of clinical phenotypes presenting either sialidosis 1 or 2 [5]. Type 1 sialidosis, the milder form, is characterized by the ocular cherry-red spots and debilitating generalized myoclonic seizures [1, 5]. Type 2 sialidosis is distinguished from type 1 sialidosis by a mucopolysaccharidosis-like figure including mental retardation, orofacial dysmorphism, kyphoscoliosis, dysostosis multiplex, recurrent pulmonary infections, pulmonary hypertension, coronary artery disease, valvular disease, cardiomyopathy, and hepatosplenomegaly [5]. For the rarity of this condition, its prevalence is uncertain.

There are only two previous reports describing anesthetic managements for patients with sialidosis [2, 3]. A 31-year-old male with type 1 sialidosis uneventfully underwent jejunostomy under spinal anesthesia [2]. A 14-year-old boy with type 2 sialidosis successfully underwent spinal column arthrodesis under general anesthesia [3]. Therefore, this is the first case report describing both general and epidural anesthesia for a patient with type 1 sialidosis.

There is only limited information available on the anesthetic management of patients with sialidosis. For the first surgery in our patient, anesthesia was planned as TIVA using propofol and remifentanil. The intelligence level of type 1 sialidosis patients ranges from normal to slightly impaired [5, 6]. Pathologically, cytoplasmic accumulation of sialyloligosaccharides has been observed in the central nervous systems and the neuroradiological imaging studies frequently reveal diffuse brain atrophy in type 1 sialidosis patients [7]. Therefore, prolonged emergence from general anesthesia might be expected in these patients, even in those with no mental retardation. It is desirable during the general anesthetic managements to use short-acting agents under close monitoring to prevent delayed emergence. Thus, multimodal analgesia might be useful. Additionally, propofol, which has an anticonvulsant effect would be suitable for patients with myoclonus. It should be noted that glycoprotein storage disorders have not been specified as being malignant hyperthermia susceptible disease [8].

For the second surgery, anesthesia was planned as a combination of general and epidural anesthesia to reduce the amount of general anesthetics and systemic opioids used. Based on the successful management with spinal anesthesia in a patient with type 1 sialidosis [2], epidural anesthesia is likely to be acceptable in these patients, depending on the situation.

As type 1 sialidosis generally does not present with orofacial deformation [5], the airway management of patients with type 1 sialidosis might not be challenging. However, due to the lack of detailed verification of the digestive function in sialidosis patients, rapid sequence induction might be preferable even in no history of accidental aspiration.

Due to insufficient verification of drug pharmacodynamics and pharmacokinetics in this disease, anesthesiologists carefully assess these patients and give careful consideration to individually tailored anesthetic managements.

## Conclusions

We uneventfully provided both general and epidural anesthesia for a patient with type 1 sialidosis. Anesthesiologists should carefully assess patients with sialidosis and should individualize the anesthetic management based on their characteristics.

## Data Availability

Not applicable

## Abbreviations

TIVA: Total intravenous anesthesia
BIS: Bispectral index
TCI: Targeted controlled infusion
PCEA: Epidural patient-controlled analgesia
TOF: Train-of-four
